# A textured clinical mosaic involving autoimmune calcific constrictive pericarditis: a case report

**DOI:** 10.1093/ehjcr/ytaf301

**Published:** 2025-07-03

**Authors:** Michele Bertelli, Luca Bergamaschi, Matteo Armillotta, Francesco Angeli, Carmine Pizzi

**Affiliations:** Cardiology Unit, Cardiac Thoracic and Vascular Department, IRCCS Azienda Ospedaliera-Universitaria di Bologna, viale Giambattista Ercolani 17, Bologna 40138, Italy; Department of Medical and Surgical Sciences—DIMEC—Alma Mater Studiorum, University of Bologna, Via Giuseppe Massarenti 9, Bologna 40138, Italy; Cardiology Unit, Cardiac Thoracic and Vascular Department, IRCCS Azienda Ospedaliera-Universitaria di Bologna, viale Giambattista Ercolani 17, Bologna 40138, Italy; Department of Medical and Surgical Sciences—DIMEC—Alma Mater Studiorum, University of Bologna, Via Giuseppe Massarenti 9, Bologna 40138, Italy; Cardiology Unit, Cardiac Thoracic and Vascular Department, IRCCS Azienda Ospedaliera-Universitaria di Bologna, viale Giambattista Ercolani 17, Bologna 40138, Italy; Department of Medical and Surgical Sciences—DIMEC—Alma Mater Studiorum, University of Bologna, Via Giuseppe Massarenti 9, Bologna 40138, Italy; Cardiology Unit, Cardiac Thoracic and Vascular Department, IRCCS Azienda Ospedaliera-Universitaria di Bologna, viale Giambattista Ercolani 17, Bologna 40138, Italy; Department of Medical and Surgical Sciences—DIMEC—Alma Mater Studiorum, University of Bologna, Via Giuseppe Massarenti 9, Bologna 40138, Italy; Cardiology Unit, Cardiac Thoracic and Vascular Department, IRCCS Azienda Ospedaliera-Universitaria di Bologna, viale Giambattista Ercolani 17, Bologna 40138, Italy; Department of Medical and Surgical Sciences—DIMEC—Alma Mater Studiorum, University of Bologna, Via Giuseppe Massarenti 9, Bologna 40138, Italy

**Keywords:** Constrictive pericarditis, Systemic lupus erythematosus, Right heart catheterization, Thoracic computed tomography, Cardiac magnetic resonance, Case report

## Abstract

**Background:**

Constrictive pericarditis is a rare condition whereby chronic pericardial inflammation leads to pericardial stiffening and predominantly right-sided heart failure. While idiopathic and infectious forms are most common, autoimmune causes may be involved with often elusive disease manifestations.

**Case summary:**

A 34-year-old woman presented with severe right-sided heart failure and atrial fibrillation secondary to rapidly progressive calcific constrictive pericarditis following preterm delivery due to placenta previa. Given the refractoriness to medical therapy and dependence on i.v. diuretic therapy, surgical pericardiectomy was performed without any clinical benefit (prompt heart failure relapse). Further tests revealed a mosaic of multiorgan manifestations (alveolar haemorrhage, pleuritis, factor XI deficiency, altered lymphocyte subpopulations, cutaneous nodules demonstrating Koebner’s phenomenon, placental vasculopathy causing miscarriage and preterm delivery), which ultimately led to the diagnosis of systemic lupus erythematosus (SLE). Instatement of immunosuppression (corticosteroid initially, subsequently substituted by mycophenolate) led to the resolution of constriction and other disease manifestations at 18-month follow-up.

**Discussion:**

This case of SLE presenting with advanced calcific constrictive pericarditis demonstrates the crucial role played by aetiological diagnosis in constrictive pericarditis. It also exemplifies the often-subtle nature of extra-cardiac manifestations in autoimmune processes, particularly in SLE, which may lead to significant diagnostic uncertainty and delay in the instatement of disease-specific therapy. The benefits of the latter are manifest in this case as the introduction of mycophenolate determined a complete reversal of constrictive physiology despite refractoriness to surgical pericardiectomy.

Learning points

- Systemic lupus erythematosus can present with rapidly progressive constrictive pericarditis alongside various subtle multiorgan manifestations.
- Instatement of disease-specific immunosuppression can provide significant haemodynamic benefit through reversal of constrictive physiology.

## Introduction

Constrictive pericarditis (CP) is a rare condition whereby chronic pericardial inflammation leads to loss of pericardial compliance and impediment of diastolic filling, thereby determining predominantly right-sided heart failure.^1^ While idiopathic and infectious forms are most common, autoimmune disease may be involved.^1^ Definitive treatment of CP is surgical pericardiectomy, which however is known to be ineffective in a proportion of patients likely owing to myocardial compliance alterations but also residual pericardial constriction.^1^ Indeed, in some cases, inflammation and constriction may occur solely in the epicardium, which is unaffected by pericardiectomy.^1^ In this setting, where pericardiectomy offers no therapeutic benefit, timely diagnosis and instatement of disease-specific medical therapy may be particularly beneficial. Nonetheless, in the case of autoimmune CP, particularly in systemic lupus erythematosus (SLE), this may be challenging due to the often-elusive multiorgan involvement. Herein, we describe the diagnostic challenges and therapeutic benefits of disease-specific immunosuppression in a case of CP secondary to SLE refractory to pericardiectomy.

## Summary figure

**Figure:**
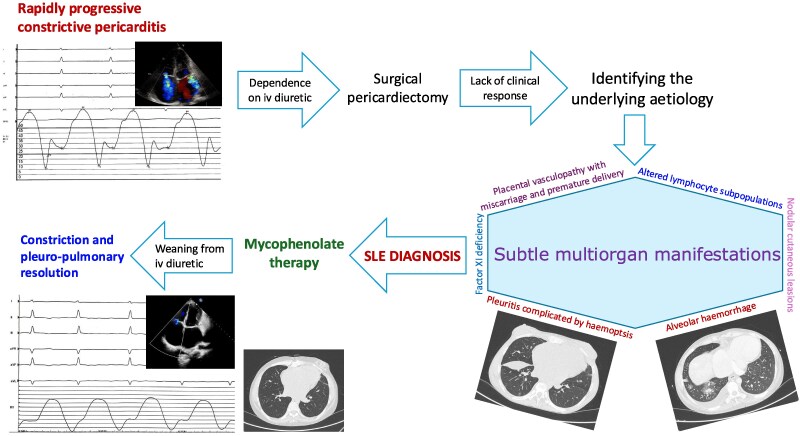


## Case presentation

**Figure 1:**
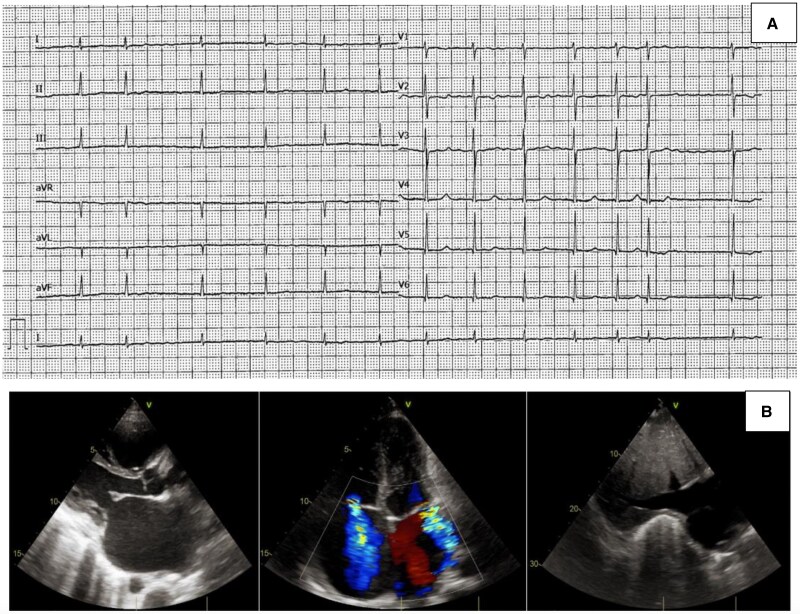
Cardiological investigations at presentation. Electrocardiogram (*A*) and echocardiogram (*B*).

A 34-year-old woman of Nigerian descent presented with new-onset atrial fibrillation and repolarization abnormalities (diffuse flat T waves) at 14-week gestation. Her past medical history included previous second-trimester miscarriage with placental infarction and fibrin deposition. Shortly after preterm delivery due to placenta previa with premature rupture of membranes, she developed dyspnoea, peripheral oedema, hepatosplenomegaly, and ascites. At presentation, vital signs were as follows: blood pressure 90/60 mmHg, heart rate 80 b.p.m., peripheral oxygen saturation 93%, and temperature 36.5°C. The echocardiogram revealed severe biatrial dilation, moderate mitral and tricuspid regurgitation, septal bounce, raised estimated systolic pulmonary arterial pressure [peak tricuspid regurgitation pressure gradient (TRPG), 26 mmHg; inferior vena cava (IVC), 25 mm with no collapsibility], and pericardial thickening [*Figure 1B*; left ventricular end-diastolic volume (LVEDV), 72 mL; left ventricular ejection fraction (LVEF), 65%; tricuspid annular plane systolic excursion (TAPSE), 11 mm; right ventricular (RV) end-diastolic area (RVEDA), 16 cm^2^; fractional area change (FAC), 34%]. Given the suspicion of CP, the patient was referred to our cardiology department.

**Figure 2:**
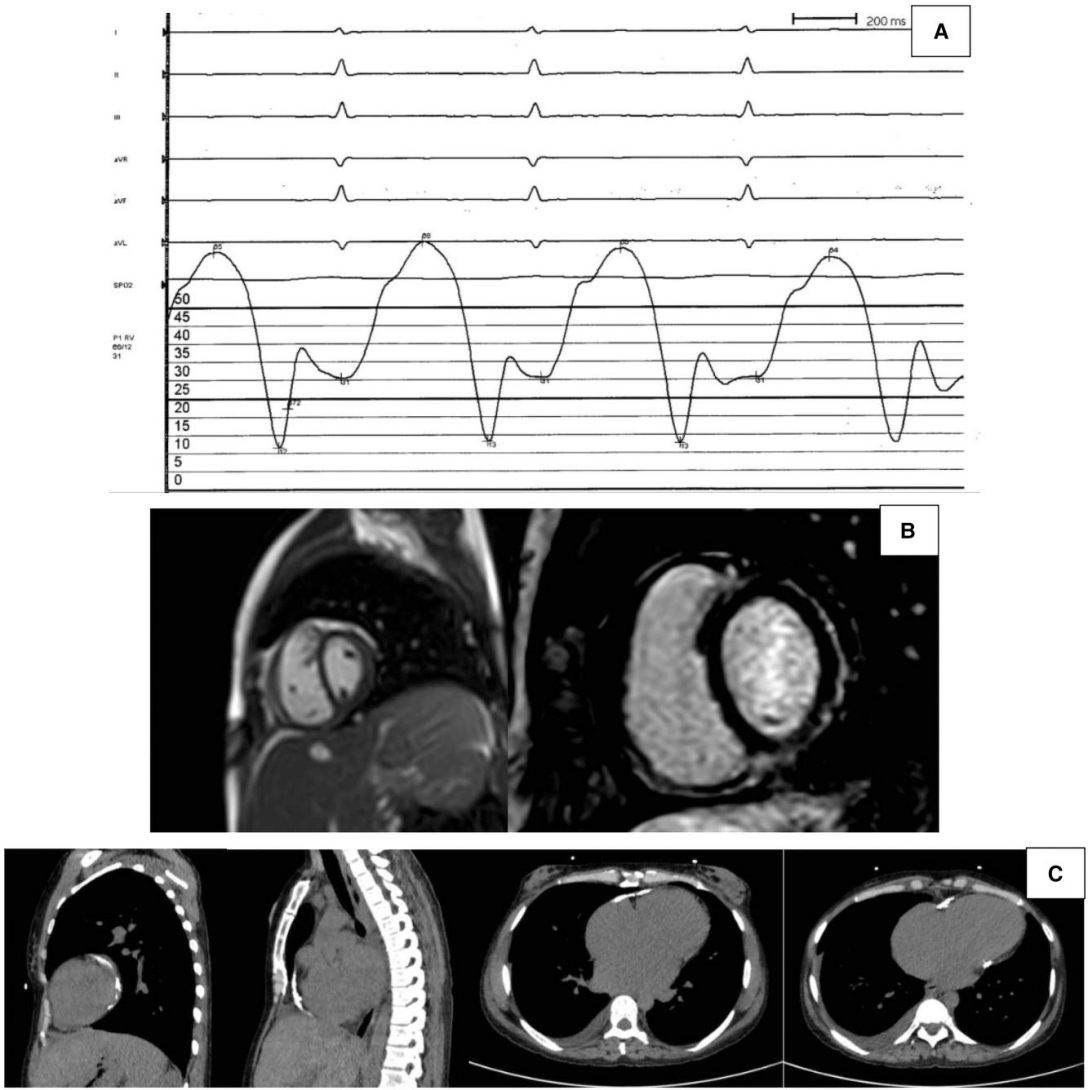
Investigations confirming calcific constrictive pericarditis diagnosis. (*A*) Right ventricular pressure curve at right heart catheterization demonstrating dip-and-plateau pattern. (*B*) Cardiac magnetic resonance showing paradoxical septal motion on cine sequence (left) and localized pericardial late gadolinium enhancement (right). (*C*) Thoracic computed tomography showing diffuse pericardial calcification.

Blood tests demonstrated lymphopenia without any rise in acute inflammatory markers (only mildly raised erythrocyte sedimentation rate). Screening of autoimmune and infectious causes of pericarditis revealed mild ANA positivity (1:80 with speckled cytoplasmic pattern). The patient underwent right heart catheterization (RHC), demonstrating dip-and-plateau pattern of RV pressure curve and equalization of pressures across the RV, right atrium, and left atrium (*Figure 2A*). Cardiac magnetic resonance (CMR) showed signs of localized pericardial but not myocardial inflammation [inferolateral pericardial late gadolinium enhancement (LGE); indexed LVEDV, 60 mL/m^2^; LVEF, 55%; indexed RV end-diastolic volume, 73 mL/m^2^; RV ejection fraction, 48%; T1 and T2 not assessable due to artefacts), while thoracic computed tomography (CT) revealed circumferential pericardial calcifications (*Figure 2B* and *C*).

**Figure 3:**
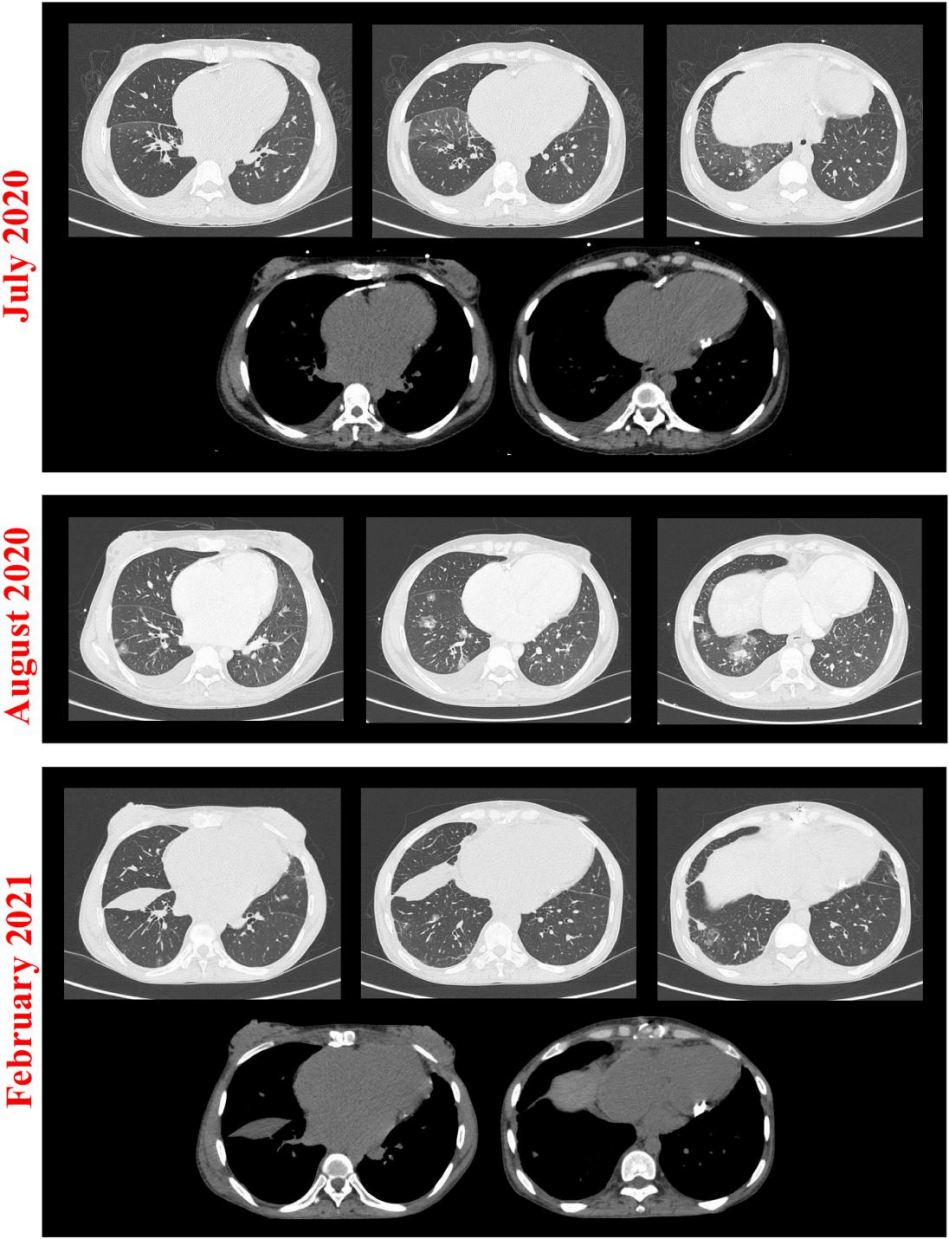
Pulmonary manifestations. Pulmonary window thoracic computed tomographies (top of image subsets) demonstrating multiple bilateral nodules migrating over time (areas of alveolar haemorrhage) and right-sided infrascissural pleural effusion (February 2021) consistent with pleuritis; mediastinal window thoracic computed tomographies (bottom of image subsets) showing pericardial calcifications pre- (July 2020) and post-pericardiectomy (February 2021).

The patient was treated with high-dose intravenous diuretic, rate control, and anticoagulation. However, any attempt to wean her from i.v. diuretics led to worsening congestion. Given the refractoriness to medical therapy, complete surgical pericardiectomy was performed. Histopathology of pericardial tissue revealed fibrosis, calcification, and lymphocyte/macrophage infiltrates without granulomas. The patient was discharged with high-dose oral diuretic but after 3 months presented with relapsing peripheral oedema, ascites, and new-onset haemoptysis. Thoracic CT showed a large right-sided haemopneumothorax (requiring pleural drainage) and basal/infrascissural pleural effusion (*Figure 3*, bottom). High-dose i.v. diuretic was reintroduced, and rate control medications were interrupted to maximize cardiac output. Further blood tests demonstrated factor XI deficiency and persistent lymphopenia, with altered lymphocyte subpopulations (reduced CD8+ effector memory, raised CD4+/CD8+ ratio and CD8+ central memory). On further examination, painful cutaneous nodules were noted at venepuncture sites. Lastly, thoracic CT images were reviewed revealing bilateral migratory nodules surrounded by ground-glass halo (*Figure 3*), which were demonstrated by CT-guided biopsy to be areas of focal alveolar damage with capillaritis and neutrophil infiltrates.

**Figure 4:**
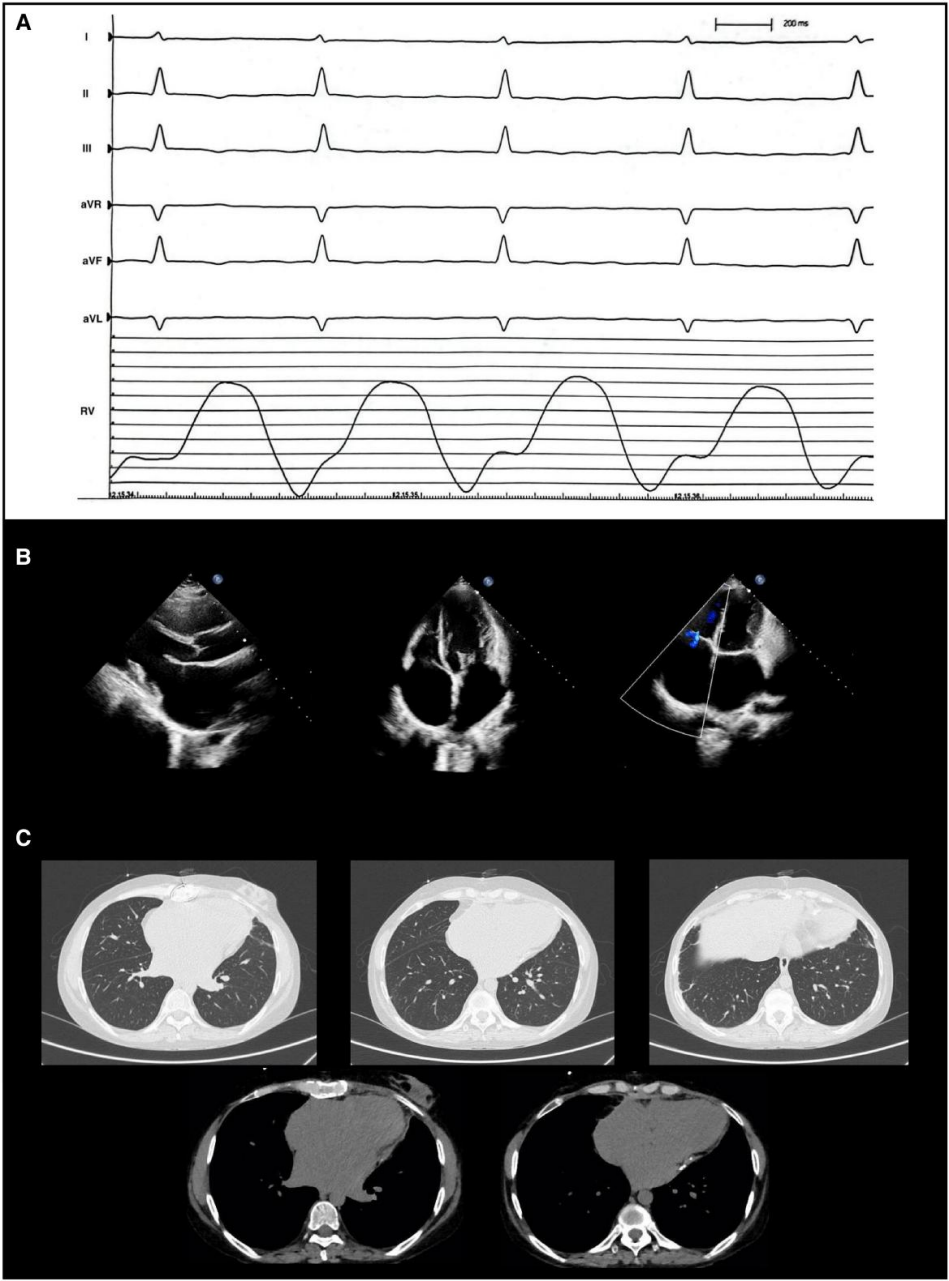
Therapeutic response to immunosuppression. Right heart catheterization (*A*), echocardiogram (*B*), and computed tomography thorax (*C*) after 18 months of mycophenolate immunosuppression. Note the normalization of right ventricular pressure curves, reduction in biatrial dilation, and mitral and tricuspid regurgitation; pulmonary window computed tomography scans demonstrate resolution of pulmonary nodules and pleural effusion (*C*, top), while mediastinal window computed tomography scans show reduction in pericardial calcifications (*C*, bottom) compared with post-pericardiectomy computed tomography scans.

Such multiorgan involvement oriented our diagnosis to SLE, as the patient met its diagnostic criteria.^2^ Furthermore, from a literature review, all signs noted in this patient emerged as potential SLE manifestations^3–13^ ; see Supplementary material section. Therefore, high-dose oral corticosteroid and, following rheumatology review, mycophenolate were introduced. The patient was discharged on oral diuretic and progressive weaning from corticosteroid until suspension. At 18 months after discharge, RHC documented no signs of constriction with mildly raised RV pressures and post-capillary pulmonary hypertension (*Figure 4A*). Echocardiography, in turn, documented a reduction in biatrial dilation and both mitral and tricuspid regurgitation (*Figure 4B*; peak TRPG 16 mmHg, IVC 20 mm with <50% collapsibility, LVEDV 67 mL, LVEF 61%, TAPSE 13 mm, RVEDA 12 cm^2^, FAC 42%). Lastly, thoracic CT showed few residual pericardial calcifications and resolution of pulmonary nodules (*Figure 4C*).

## Discussion

This case constitutes an example of SLE presenting with severe calcific CP and a wide range of multiorgan manifestations: pleuritis complicated by haemopneumothorax, diffuse alveolar haemorrhage, factor XI deficiency, altered lymphocyte subpopulations, miscarriage with placental vasculopathy/fibrin deposition, and subacute nodular cutaneous lesions. It exemplifies how the subtle nature of SLE’s multiorgan manifestations may complicate diagnosis delaying the introduction of targeted therapy. Beyond CP, in this case, pleuro-pulmonary involvement was a predominant feature and one that oriented our ultimate aetiological diagnosis.^3^

Regarding the disease progression observed, the subtle immunological alterations without raised acute inflammatory markers suggest a subacute phase of disease at presentation. This may indicate that pericardial inflammation had been ongoing for sufficient time to cause diffuse calcification and is confirmed by the limited pericardial LGE on CMR. The interaction with recent pregnancy further strengthens this assertion given that this is associated with increased risk of flares in active SLE.^14^ Early during her second pregnancy, atrial fibrillation was present, likely indicating ongoing pericardial inflammation. At the time of our first assessment (2 weeks after delivery), in turn, inflammatory markers were normal, and severe constriction and calcification were present. Such disease progression is in keeping with the rapid progression of pericardial inflammation to constriction in SLE as already described in previous case reports.^4,5,7^

In terms of the therapeutic response observed in this patient, two observations can be drawn. Firstly, the lack of response to complete pericardiectomy may be explained by residual epicardial inflammation,^1^ which may have been missed by CMR LGE sequences. Secondly, the introduction of immunosuppression with mycophenolate appeared to completely reverse constriction. Such haemodynamic response may be explained by reversal of residual epicardial inflammation and calcification by mycophenolate, an analogous phenomenon having been described in a case of calcinosis cutis universalis.^15^ Although more work on the immunomodulatory effects on pleuro-pericardial tissue by mycophenolate is warranted, its use may be considered in patients with autoimmune calcific CP refractory to pericardiectomy.

## Conclusions

This case represents an example of SLE with multiorgan involvement presenting with advanced calcific CP, refractory to surgical pericardiectomy but responsive to disease-specific immunosuppression. When confronted with non-infectious calcific CP, autoimmune aetiologies and in particular SLE should be suspected. In this setting, mycophenolate may prove beneficial through reversal of inflammatory pericardial constriction.

## Lead author biography


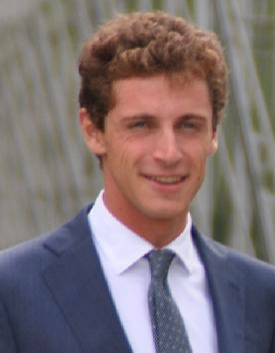


Michele Bertelli is a cardiologist with a specific interest in heart failure and electrophysiology. He received his medical degree at University College London (UK) in 2019 and trained in cardiology at Bologna University Hospital (Italy) between 2021 and 2024. He is currently working as an Advanced Heart Failure fellow at the University of Calgary (Canada).

## Supplementary Material

ytaf301_Supplementary_Data

## Data Availability

The data underlying this article will be shared on reasonable request to the corresponding author.

## Supplementary material

**Consent:** Written consent for submission and publication of this case report has been obtained from the patient in line with COPE guidelines.

**Funding:** None declared.
