# Supplementary material for: A textured clinical mosaic involving autoimmune calcific constrictive pericarditis: a case report
Source: Eur Heart J Case Rep. 2025 Jul 3;9(7):ytaf301. doi: 10.1093/ehjcr/ytaf301 (PMC12247502; doi:10.1093/ehjcr/ytaf301)
Supplement: ytaf301_Supplementary_Data [file ytaf301_supplementary_data.zip › Supplementary material - Table 1.docx]

**Supplementary material**

**Table 1**

Studies reporting the clinical manifestations observed in this patient as disease expressions of SLE.

|  | **Authors** | **Journal** | **Type of article** | **Synopsis** |
| --- | --- | --- | --- | --- |
| ***Constrictive pericarditis*** | Pieta A et al. 2021 (4) | *Rheumatol Int* | Case report | Calcified CP resulting in tamponade |
|  | Gupta S et al. 2020 (5) | *Cureus* | Case report | Rapidly progressing CP as initial manifestation of SLE |
|  | Oh JY et al. 2012 (7) | *Eur Heart J Cardiovasc Imaging* | Case report | CP with pericardial LGE on CMR and clinical response to corticosteroid therapy |
|  | Starkley RH et al. 1973 (8) | *Chest* | Case report | Rapid development of CP following acute pericarditis |
| ***Pleuritis and alveolar haemorrhage*** | Shin JI et al. 2022 (2) | *J Clin Med* | Review | Lupus pleuritis may present with haemopneumothorax; ground-glass opacities observed in pulmonary involvement; biopsy of diffuse alveolar haemorrhage typically demonstrates capillaritis and neutrophil infiltrate |
| ***Placental lesions*** | Dhital R et al. 2014 (9) | *Rheumatology* | Retrospective study | Perivillous fibrin deposition most common finding in placenta of SLE patients and associated with small for gestational age infants |
| ***Factor XI deficiency*** | Bortoli R et al. 2008 (10) | *Semin Arthritis Rheum* | Case report and literature review | Acquired XI deficiency in recent SLE diagnosis presenting with life-threatening bleeding; review of other 13 cases (8 with bleeding events) |
| ***Lymphocyte subpopulation alterations*** | Kandane-Rathnayake R et al. 2021 (11) | *Rheumatology* | Prospective study | 18287 patients; lymphopenia present in 37.2% and associated with disease activity |
|  | Fritsch RD et al. 2006 (12) | *Arthritis Rheum* | In vitro phenotypic analysis | Flow cytometry on 37 patients; increased terminally differentiated CD4+ cells with increased cycling and apoptosis |
|  | Chen P-M et al. 2021 (13) | *Current Opinion in Rheumatology* | Review | Reduced CD8+ function involved in poor peripheral immunological tolerance in SLE |
| ***Koebner phenomenon*** | Ueki H et al. 2005 (14) | *Autoimmunity Review* | Review | Koebner phenomenon expression of discoid lupus and arises from various |
